# Evaluation of the effect of curcumin and zinc co-supplementation on glycemic measurements, lipid profiles, and inflammatory and antioxidant biomarkers in overweight or obese prediabetic patients: a study protocol for a randomized double-blind placebo-controlled phase 2 clinical trial

**DOI:** 10.1186/s13063-020-04923-w

**Published:** 2020-11-30

**Authors:** Majid Karandish, Hassan Mozaffari-khosravi, Seyed Mohammad Mohammadi, Maryam Azhdari, Bahman Cheraghian

**Affiliations:** 1 Nutrition and Metabolic Diseases Research Center, Ahvaz Jundishapur University of Medical Sciences, Ahvaz, Iran; 2 Department of Nutrition, School of Public Health, Shahid Sadoughi University of Medical Sciences, Yazd, Iran; 3 School of Medicine, Shahid Sadoughi University of Medical Sciences, Yazd, Iran; 4 Student Research Committee, Ahvaz Jundishapur University of Medical Sciences, Ahvaz, Iran; 5 Department of Nutrition, School of Allied Medical Sciences, Ahvaz Jundishapur University of Medical Sciences, Ahvaz, Iran; 6 Department of Biostatistics and Epidemiology, School of Health Sciences, Ahvaz Jundishapur University of Medical Sciences, Ahvaz, Iran

**Keywords:** Curcumin, Zinc, Prediabetes, Glycemic measurement, Lipid profile, Antioxidant biomarkers, Inflammatory biomarkers, Randomized controlled trial

## Abstract

**Background:**

The prevalence of prediabetes is increasing worldwide. Unfortunately, prediabetes is related to non-communicable diseases. A high risk of developing type 2 diabetes mellitus (T2DM) is reported in people with prediabetes. Curcumin, a polyphenol, might lead to its therapeutic role in obesity and some obesity-related metabolic diseases. Zinc is a trace element that plays a key role in the synthesis and action of insulin, carbohydrate metabolism, and decreasing inflammation. There has been no clinical trial of zinc and curcumin co-supplementation in patients with prediabetes. In previous studies, the single administration of zinc or curcumin has not been conducted on many of the studied markers in prediabetic patients.

**Methods:**

The purpose of this randomized double-blind placebo-controlled clinical trial is to investigate the effect of curcumin and zinc co-supplementation on glycemic measurements, lipid profiles, and inflammatory and antioxidant biomarkers among 84 prediabetic patients with body mass index (BMI) between 25 and 35. Also, liver enzyme, serum zinc, urine zinc, blood pressure, anthropometric parameters, quality of life, adherence to co-supplementation, the side effects of co-supplementation, physical activity, and dietary intake will be assessed. Women or men (18–50 years old for men and 18 years to before menopause for women) will be followed for 3 months (90 days). This study will be conducted at Yazd Diabetes Research Clinic, Shahid Sadoughi University of Medical Sciences.

**Discussion:**

A diet rich in antioxidants, polyphenols, and phytochemicals has been shown to have a beneficial role in prediabetes. According to the beneficial properties of curcumin or zinc and inadequate evidence, RCTs are needed to assess the effect of curcumin and zinc co-supplementation in native prediabetes patients. We hope the results of the present trial, negative or positive, fill this gap in the literature and facilitate the approach for a much larger, multi-center clinical trial. In conclusion, a synergic effect of co-supplementation along with a weight-loss diet may delay the progression to type 2 diabetes mellitus.

**Trial registration:**

Iranian Registry of Clinical Trials (IRCT) IRCT20190902044671N1. Registered on 11 October 2019

## Introduction

### Background and rationale

Prediabetes or intermediate hyperglycemia [1] is the state referred to as impaired fasting glucose (IFG), impaired glucose tolerance (IGT), or glycated hemoglobin A1C (HbA1C) of 5.7 to 6.4% (39–47 mmol/mol). There is a difference in terms of the diagnostic criteria for illustrating a prediabetes state between the American Diabetes Association (ADA) [2], International Diabetes Federation (IDF) [3], and World Health Organization (WHO) [4] (Supplementary Material, Table S1). The prevalence of prediabetes is increasing worldwide [5].

Unfortunately, prediabetes is related to non-communicable diseases (the different types of obesity, dyslipidemia, and hypertension) [6]. A high risk of developing type 2 diabetes mellitus (T2DM) is reported in people with prediabetes [7]. However, it is preventable [8] by lifestyle interventions [9] (such as weight-loss strategies using dietary changes and/or physical activity [10]). The beneficial effects of a diet rich in antioxidants [11], polyphenols [12], and phytochemicals [13] have been shown in many studies.

Turmeric (*Curcuma longa*) is a plant-derived spice related to the ginger family (Zingiberaceae) with medicinal properties. The largest producer of turmeric is India. The bioactive yellow molecules in turmeric are called curcuminoids [14] that consist of curcumin (diferuloylmethane) (CUR), demethoxycurcumin (DMC), and bisdemethoxycurcumin (BMC) [15]. Curcuminoids are generally recognized as safe (GRAS) according to the US Food and Drug Administration (FDA) [16]. Also, a maximum single oral dose (12 g/day) of curcuminoids is well tolerated [17]. In addition, the 6-month intervention of curcumin shows only slight adverse effects [18]. Curcumin, a polyphenol [19], can improve metabolic syndrome (MeS) [20], diabetes, antioxidant capacity [21], cancer [15], and arthritis [22].

Curcumin modulates the several cellular transduction pathways and molecular targets (advanced glycation end-product (AGE)-mediated induction of the receptor for AGE gene expression, de novo synthesis of glutathione, PPARγ activity, NF-kB, STAT-3, Nrf2, TNF-α, IL-1β, resistin and leptin, adiponectin, etc.) which might lead to its therapeutic role in obesity and some obesity-related metabolic diseases such as T2DM [23–25]. The effect of curcumin alone or combined with nutraceuticals in prediabetic patients was shown in three trials [26–28]. In four studies, it was assessed in the patients with MeS (prediabetes, pre-hypertension, or dyslipidemia) [29, 30] and non-alcoholic fatty liver disease (NAFLD) [31] or prediabetic or controlled T2DM [32].

Zinc is a trace element that plays a key role in more than 300 enzymes [33] such as antioxidant enzymes, synthesis and action of insulin, carbohydrate metabolism [34], and decreasing inflammation [35]. Zinc levels improve the glycemic status, lipid parameters [36] (total cholesterol (TC), serum low-density lipoprotein cholesterol (LDL-C), and triglycerides (TG)) [37], and blood pressure (BP) [36]. Some complications of diabetes may be related to oxidative stress, and zinc can be an essential element in the cellular antioxidative defense [34]. Also, there is a high concentration of zinc in human pancreatic beta cells [38]. The effect of zinc alone or combined was assessed in prediabetic patients by two trials [39, 40], and it was investigated on MeS only in one trial [41].

The characterizations of the previous studies, which are related to the title of this research, are shown in Table S2 (Supplementary Material).

Both zinc and curcumin have antioxidant properties [21, 34]. Co-supplementation of curcumin and zinc in patients with prediabetes is not yet studied. Also, in previous studies, the effect of a single administration of zinc or curcumin has not been evaluated simultaneously on many of the studied markers in patients with prediabetes. These supplementations are remarkably free of toxicity [19, 42], and they have been used as an available and inexpensive food condiment for human consumption. It is proposed that their usage along with a weight-loss diet may show synergistic effects in prediabetes. So, this double-blind, randomized placebo-controlled clinical trial (randomized clinical trial (RCT)) will start with the following objectives and hypothesis.

#### Primary objective

The primary objective is to compare the mean changes in the serum levels of biochemical parameters (fasting plasma glucose (FPG), 2 h post-prandial (2hpp), HbA1c, serum insulin, insulin resistance (IR), insulin sensitivity (IS) %, beta cell function (BCF) %, TG, TC, LDL-C, high-density lipoprotein (HDL), very low-density lipoprotein (VLDL), total blood antioxidant capacity (TAC), malondialdehyde (MDA), serum zinc, urine zinc, serum interleukin-B (IL-1B), and high-sensitivity C-reactive protein (hs-CRP)) between the groups.

#### Secondary objective

The secondary objective is to compare the mean changes of anthropometric data (weight, height, waist circumstance (WC), hip circumstance (HC), body mass index (BMI), waist-height ratio (WHtR), waist-hip ratio (WHR), fat mass (FM), free fat mass (FFM), muscle mass (MM), and A Body Shape Index (ABSI)), physical activity (PA), diastolic blood pressure (DBP), systolic blood pressure (SBP), serum alanine transaminase (ALT), serum aspartate enzyme transaminase (AST), health-related quality of life (HRQOL), and dietary intake between the groups.

### Hypothesis

Curcumin and zinc co-supplementation will significantly improve the serum levels of biochemical parameters, anthropometric data, HRQOL, PA, and BP in prediabetic patients.

### Trial design

We designed a single-center, double-blinded, randomized placebo-controlled clinical trial among patients with prediabetes with a convenience sampling technique.

## Method

### Study design

**Fig. 1:**
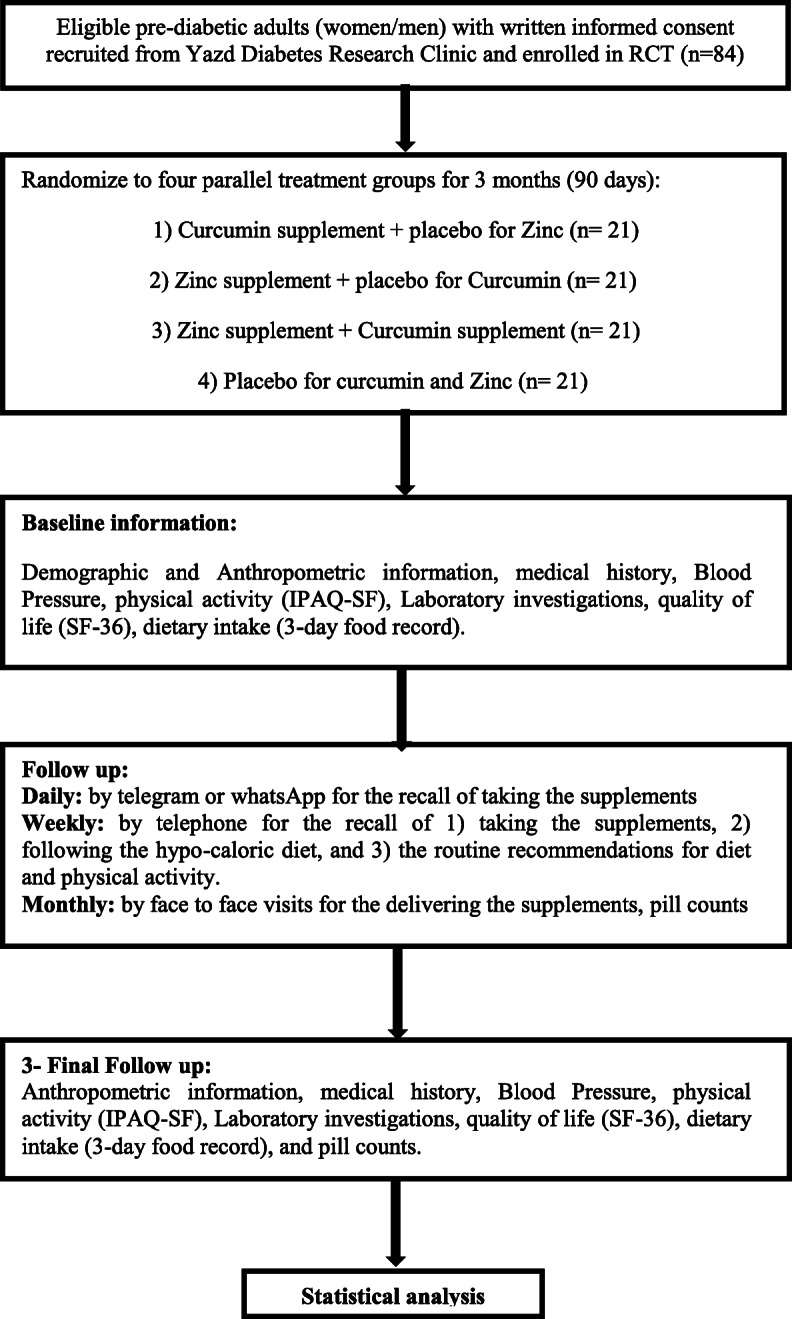
Study design flow chart. RCT, randomized controlled trial; IPAQ-SF, Short Form of International Physical Activity Questionnaire; SF-36, Short Form 36 Questionnaire

**Fig. 2:**
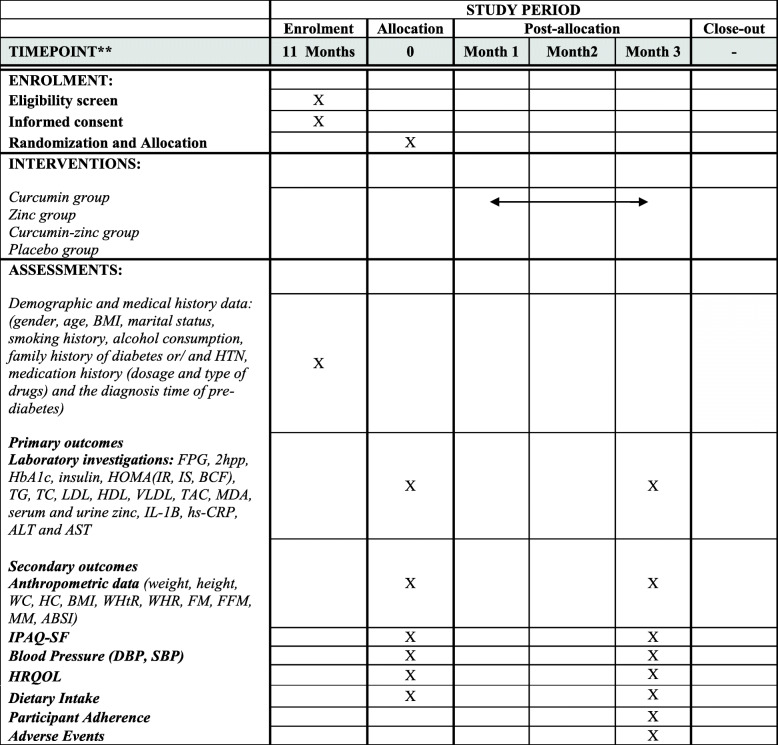
Schedule of enrollment, interventions, and assessments. ABSI, A Body Shape Index; AST, aspartate aminotransferase; ALT, alanine aminotransferase; BCM, beta cell function; BMI, body mass index; DBP, diastolic blood pressure; FFM, free fat mass; FM, fat mass; FPG, fasting plasma glucose; HC, hip circumference; HbA1c, hemoglobin A1c; HDL, high-density lipoprotein cholesterol; HOMA, homeostasis model of assessment; HRQOL, health-related quality of life; hs-CRP, high-sensitivity C-reactive protein; IL-1B, interleukin-B; IPAQ-SF, Short Form of International Physical Activity Questionnaire; IR, insulin resistance; IS, insulin sensitivity; LDL, low-density lipoprotein cholesterol; MDA, malondialdehyde; MM, muscle mass; HTN, hypertension; SBP, systolic blood pressure; TAC, total blood antioxidant capacity; TTC, total cholesterol; TG, triglycerides; VLDL, very low-density lipoprotein; WC, waist circumference; WHR, waist-hip ratio; WHtR, waist-height ratio; 2hpp, 2 h post-prandial

The flow chart of the study design and the schedule of enrollment, interventions, and assessments are shown in Figs. 1 and 2, respectively. The participant will be randomized in a 2 × 2 factorial design into four parallel treatment groups: (1) curcumin group (Cur/P.Zn), curcumin supplement + placebo for zinc; (2) zinc group (Zn/P.Cur), zinc supplement + placebo for curcumin; (3) zinc + curcumin group (Zn/Cur), zinc supplement + curcumin supplement; and (4) placebo group (P.Zn/P.Cur), placebo for curcumin and zinc. The follow-up duration will be 3 months (90 days).

This trial dated 05 October 2019 was approved by the Medical Ethics Committee of Ahvaz Jundishapur University of Medical Sciences, Ahvaz, Iran (ethical code: IR.AJUMS.REC.1398.504; available on site: http://ethics.research.ac.ir/IR.AJUMS.REC.1398.504), and it is a prospectively registered clinical trial (registration number: IRCT20190902044671N1, Iranian Registry of Clinical Trials (IRCT), available on site: https://en.irct.ir). The study will be reported according to the Consolidated Standards for Reporting Trials (CONSORT) statement (http://www.consort-statement.org/, accessed 14 April 2017). The present study will be conducted at the Diabetes Research And Clinical Practice Center of Yazd, Central Iran, where the prevalence rate of diabetes is higher than in other parts of the world [43].

### Participants

The subjects are introduced to the trial, and those who are interested to know more are invited via email, phone call, SMS, or social media (Telegram or WhatsApp) to the study center, where the study investigator explains the study in detail to the subjects. After the clarifications have been provided, the subject will be asked to voluntarily sign the consent form (Supplementary Material, Table S3), and the first appointment will be booked. In the first appointment, the consented subjects undergo screening procedures for the study under an endocrinologist according to the ADA guidelines. The eligible subjects will be enrolled in the trial and randomized to the appropriate study groups. The screening period is planned for 12 months and will continue until 84 participants are randomized in the study.

### Inclusion/exclusion criteria

#### Inclusion criteria

Women or men (18–50 years old for men and 18 years to before menopause for women) with prediabetes according to the ADA guidelines [2] (FPG ≥ 100 and < 126 mg/dl, IGT ≥ 140 and < 200 mg/dl, 2-h plasma glucose concentration after a 75-g glucose load on the oral glucose tolerance test (OGTT) ≥ 140 and < 200 mg/dl, or/and HbA1c 5.7–6.4%) and BMI between 25 and 35. Voluntary written informed consent for all patients is mandatory before any study-related procedures.

#### Exclusion criteria

The participants must not have any of the following: a diagnosis of any types of malignancies and cancers, chronic or acute hepatic disorders (hepatitis B and C, etc.), bile disease, autoimmune diseases, neurological diseases (such as epilepsy), neurological diseases, effective hereditary disorders of the liver (iron and copper storage disease), endocrine diseases (hypothyroidism, hyperthyroidism, and Cushing’s syndrome), inflammatory diseases (rheumatoid arthritis), hypertension, cardiovascular, acute or chronic kidney disease, and lung disease; taking blood pressure-, glucose-, or lipid-lowering drugs (e.g., insulin, metformin, Glucophage, or atorvastatin); taking multivitamin-mineral supplements for 3 months before or during the intervention; a history of weight loss surgery in the last year and a weight-loss plan in the last 3 months; receiving a weight-loss medicine or program; lactating, pregnant, or planning to get pregnant; a history of any condition that the investigator may consider a contraindication to participation (such as the sensitivity to supplementation or simultaneously participating in another project); unwillingness to continue cooperation, non-compliance during the intervention (compliance less than 80%); or no signed informed consent.

### Sample size

The sample size was determined based on the ability to detect a mean effect of FPG between the supplement and placebo groups for each supplement (curcumin or zinc), separately.

By using the following formula, the maximum sample size for curcumin group based on the trial by Chuengsamarn et al. [26] and zinc group based on the trial by Ranasinghe et al. [40] was determined to be 8 (${\overline{X}}_1$ = 3.64, ${\overline{X}}_2$ = − 7.54, *S*_1_ = 8.17, and *S*_2_ = 7) and 19 (${\overline{X}}_1$ = + 16, ${\overline{X}}_2$ = + 3, *S*_1_ = 12.8, and *S*_2_ = 15.3), respectively (*α* = 0.05, significance; *β* = 0.2, power). Then, the maximum sample size was considered as the final sample size, which is sufficient to evaluate the main effects. According to an expected 10% loss at follow-up, 84 subjects (21 participants in each group) will be recruited, finally.

$$ {n}_1=\frac{\left({S}_1^2+{S}_2^2\right){\left({Z}_{1-\frac{\alpha }{2}}+{Z}_{1-\beta}\right)}^2}{{\left({\overline{X}}_1-{\overline{X}}_2\right)}^2} $$

In the formula above, *S*^2^ and $\overline{X}$ show the variance and the mean effect for FBS in the placebo and treatment groups, respectively. Also, for a more accurate calculation of the sample size, the WinPepi statistical program (version 11.4: Abramson, 2011) was performed.

### Randomization and blinding

The randomization of the participants for this study will be conducted using the method of block randomization with a block size of 4 using a computer-generated random number sequence by one of the research staff (who is an independent statistician and not available to the study investigators or the study staff). Then, the allocation concealment will be conducted assigning the unique codes in order to prevent “selection bias,” and it will be maintained by the pharmacist who is a research staff not involved during the study (the pharmacist is involved only with the dispensing of the investigational products). After checking the inclusion and exclusion criteria and the baseline measurements by two investigators, the eligible participants will be given consecutive numbers, which will be forwarded to the pharmacist who will dispense the interventional product. The pharmacist will open the sealed envelope (that is given by the independent statistician containing the treatment code) after the final data analysis and/or an emergency situation. All the assessments in the trial will be made by the investigators blinded to the treatment allocation. In the event of an emergency situation where being aware of the treatment allocation is critical, the pharmacist will be accessed to unblinding envelopes provided for each participant. Further assessment and required medical or legal interventions will be carried out according to the case. The emergency unblinding and the reason should be recorded in the case report form. The treatment code would be communicated to the medical personnel in charge of the treatment but should not be recorded or verbally disclosed in any of the study documents or to the patient. Since this is a double-blinded study, the investigators, participants, and those involved in the study will not be informed about the type of supplement used except the research staff. The supplements will be delivered to the participants according to the allocation on the 1st, 30th, and 60th days.

### Intervention

The dose of supplements was calculated based on the data from the previous studies [26–32, 39, 40, 44]. A dose of 30 mg/day of zinc as zinc gluconate for 3 months will be prescribed. A dose of 30 mg/day zinc element as zinc sulfate significantly improve FPG, BCF, IS, and IR for 6 months, whereas 20 mg/day of zinc element had a favorable effect on FPG, OGTT, IR, TC, LDL-C, and BCF but for 12 months. Also, the cytotoxicity of zinc gluconate was less than that of zinc sulfate. The effect of curcumin extract on the patients with IFG, IGT, and MeS has been seen with different doses ranging from 20 to 2400 mg and follow-up duration between 2 and 9 months.

Zinc supplement contains 30 mg zinc as zinc gluconate. The curcumin supplement (BCM95/Curcugreen) used in the study is an extract of dried turmeric rhizomes which was identified by a qualified botanist as *Curcuma longa*, and a voucher specimen is kept with herbarium ID HERB-ED-22. The turmeric rhizomes were extracted with ethyl acetate, and each 500-mg capsule of curcumin supplement contained 475 mg of curcuminoids and turmeric essential oil. The purity of the bioactives in the capsule was tested by HPLC, residual solvents by GCHS, and heavy metals by ICPMS, and microbial parameters conformed to the EU standards. Placebos are identical in texture and appearance to its active supplement. The placebo for zinc is made of lactose (that was produced in the School of Pharmacy, Shahid Sadoughi University of Medical Sciences and Health Services, Iran), and the placebo capsules for curcumin are made of roasted rice powder (that was produced by “M/s Arjuna Natural Pvt Ltd., India.” All participants will receive a tablet (zinc supplement or placebo for zinc) to be consumed before breakfast and a capsule (curcumin supplement or placebo for curcumin) to be taken after breakfast, daily. Also, the energy requirement of the participants will be calculated by the report of a Joint FAO/WHO/UNU Expert Consultation [45], individually. Given that the participants are overweight or obese, they will receive a standard hypo-caloric diet with at least a 7% weight loss [46] including 45–55%, 25–35%, and 10–20% of their calories from carbohydrate, fat, and protein, respectively [47].

In each visit (visit 1, 1st day; visit 2, 30th day; visit 3, 60th day), the health information (diet and physical activity) will be recommended to all participants to improve healthy habits, individually.

The recommendations will include (1) following a hypo-caloric diet, (2) avoiding excessive intake of high-fat products (whole dairy products, poultry fat (the skin of chicken), red meat, butter, cream, ice cream, processed meats (sausages), coconut oil, and palm oil), (3) avoiding excessive sugar consumption (candy, soda, syrups, sugar-loaded desserts, caramels, and chocolate), and (4) increasing PA by walking or cycling for 150 min per week [46].

The recall of the supplement intake and the routine recommendations will be given using messaging tools (Telegram and WhatsApp) daily; the telephone service will be used for the participants who are unable to get access to these tools, weekly. If supplement intake is missed in the morning, patients will be advised to take them during the same day.

### Outcomes

The primary outcomes include serum levels of biochemistry parameters except ALT and AST. The secondary outcomes are ALT and AST, body composition, PA, BP, HRQOL, and dietary intake. The primary and secondary outcomes will be conducted at the baseline and the end of the study (day 90 after intervention) for all participants. Only adherence will be checked at the end of every month.

### Outcome measurements

All outcomes and time points are specified in Fig. 2.

### Demographic and medical information

The demographic and medical history data include gender, age, full address, postal code, marital status, income, occupation, ethnicity, educational background, smoking history, alcohol consumption, family history of diabetes or/and hypertension, medication history (dosage and type of drugs), and the diagnosis time of prediabetes.

### Anthropometric data

Weight and height will be measured using a pre-calibrated electronic scale (Seca, Germany) to the nearest 0.5 kg and a wall-mounted stadiometer to the nearest 0.1 cm, respectively. BMI (kg/m^2^) will be calculated by the following equation: weight (kg)/height (m^2^). A non-elastic and flexible tape will be used to measure WC (at the midpoint between the iliac crest and the rib cage in a standing position at the end of normal expiration) and HC (the level of the greater trochanters) to the nearest 0.1 cm.

WHtR and WHR will be calculated by dividing WC by height and WC by HC, respectively. The ABSI was calculated using the following formula: ABSI = WC (m)/(BMI^2/3^ × height^1/2^ (m)) [48].

Body composition will be estimated using the bioelectrical impedance analysis (BIA) method by a body composition analyzer (InBody 270, Seoul, South Korea).

All anthropometric assessments will be conducted by a trained assessor in duplicate, with the mean measurement recorded.

### Blood pressure

According to the American Heart Association protocol, BP will be measured at the baseline and at the end of the study by an assessor under the following conditions:

After 5 min of rest in a quiet place, the participants will be sitting without crossed legs and unsupported back and arms. The BP of the participants will be measured in both arms by the Korotkoff sound technique with a calibrated mercury sphygmomanometer (Omron, Tokyo, Japan). If a consistent difference in BP measurement between the arms is shown, the maximum pressure will be recorded. The mean of three readings will be recorded (1-min interval between them) [49].

### Laboratory investigations

Blood samples (12 ml) of all participants will be collected after 12 h of overnight fasting at the baseline and at the end of the study. The samples will be collected into two different tubes (in the tube without anticoagulant and in EDTA tubes) and centrifuged to obtain the serum or plasma. The serum samples (3 ml) will be stored at − 80 °C. The plasma and the remaining serum will be used to determine the FPG, 2hpp (glucose oxidase/peroxidase method, BioSystem, Spain), HbA1c (HPLC, TOSOH, Japan), TG (glycerol phosphate oxidase/peroxidase method, BioSystem, Spain), TC (cholesterol oxidase/peroxidase method, BioSystem, Spain), HDL-C (direct method, BioSystem, Spain), SOD (colorimetric (420 nm) method, ZellBio GmbH, Germany), TAC (colorimetric (570 nm) method, ZellBio GmbH, Germany), MDA (colorimetric (535 nm) method, ZellBio GmbH, Germany), ALT, AST (IFCC method, BioSystem, Spain), serum insulin (enzyme-linked immunosorbent assay (ELISA) kit, Monobind, USA), serum zinc (flame atomic absorption spectrometry method (Ziest Chem Diagnostics kit, Iran), serum IL-1B (human IL-1β (ELISA kit), BE58011, IBL, Hamburg, Germany), and serum hs-CRP )ELISA kit, EU59131, IBL, Hamburg, Germany). At least 1 ml of random urine specimen (the fasting state is not necessary for urine collection) will be taken at the baseline and at the end of the study for the measurement (urine zinc by flame atomic absorption spectrometry method (Ziest Chem Diagnostics kit, Iran; normal range 15–150 μg/dl). All biochemical tests except IL-1B, hs-CRP, SOD, TAC, and MDA will be conducted immediately after sampling.

The following formula will be used to calculate VLDL and LDL-C:

- VLDL = TG (mg/dl)/5 (this formula is valid only when TG are ≤ 400 mg/day)
- LDL-C = TC (mg/dl) − HDL-c (mg/dl) − TG (mg/dl)/5; Friedewald formula [50].

The homeostasis model of assessment 2 (HOMA-2) calculator will be downloaded from the University of Oxford and used to calculate IR, S%, and BCF% (http://www.dtu.ox.ac.uk/).

### Dietary intake

Dietary intake will be recorded using 3-day food intake records (2 weekdays, 1 weekend day), which is a validated tool for diet analysis [51], at the baseline and at the end of the study by the participants (self-administered). The dietitian will train the participants to record the amount of food consumed with multiple homemade cutlery in order to enhance the accuracy of the portion size and review all entries in the food records. A blinded expert dietitian to the study allocation will review all forms of the completed 3-day food intake records. Dietary intake data will be analyzed using the Nutritionist IV software (version I) to estimate the energy and the number of macronutrients.

Also, they will be qualitatively recommended the dietary intake to improve dietary habits by messaging tools or telephone, weekly. The qualitative dietary recommendations will include (1) follow her/his diet, (2) avoid excessive intake of high-fat products (whole dairy products, poultry fat (the skin of chicken), red meat, butter, cream, ice cream, processed meats (sausages), coconut oil, and palm oil), and (3) avoid excessive sugar consumption (candy, soda, syrups, sugar-loaded desserts, caramels, and chocolate).

### Physical activity

The Short Form of International Physical Activity Questionnaire (IPAQ-SF), acceptable reliability and validity, will be used to assess total weekly PA [52] by a face-to-face interview. The IPAQ is suitable for adults between 15 and 69 years of age. IPAQ-SF includes four generic items (walking, moderate (such as leisure cycling), vigorous activities (such as aerobics), and sitting) and reported as minutes per week (min/week) within each activity category by a metabolic equivalent of task (MET) energy expenditure.

The following formula will be used to calculate the PA (MET min week^−1^):

$$ \mathrm{MET}\ \mathrm{level}\times \mathrm{duration}\times \mathrm{frequency}\ \mathrm{per}\ \mathrm{week}. $$

The results of PA will be categorized into 3 levels (vigorous-intensity activity, moderate-intensity activity, low-intensity activity) [53]. The participants will be interviewed by the blinded research staff to the study allocation to fill IPAQ-SF.

### Health-related quality of life

A validated and translated to Persian Short Form Health Survey (SF-36) questionnaire will be used to assess health-related quality of life (HRQOL) [54]. The assessment of HRQOL will show the patients’ overall health status, the impact of treatment, the formulation of health policy, and the decision on resource allocation [55]. Eight health-related dimensions will be measured by the SF-36 questionnaire that includes physical functioning (10 items), role limitations due to physical problems (4 items) and emotional problems (3 items), bodily pain (2 items), general health perceptions (5 items), vitality (4 items), social functioning (2 items), and perceived mental health (5 items). Also, there is a single item for health transition that provides an indication of perceived change in general health status over a 1-year period. The score of SF-36 will be obtained from the sum of the questions in eight domains. Scores range from 0 to 100. The participants with more or less disability will have lower or higher scores of SF-36, respectively [56].

### Participant adherence

At each follow-up visit, (1) participants will be briefed on the study guidelines (dietary and PA recommendation), adherence to the dosing schedule, dose timing, storage, and missed dose; (2) participants will be asked to return the unused supplementation and bottle; and (3) the importance of contacting the study assessor will also be noted in case of any problems (e.g., unusual symptoms). Adherence to the intake of the supplements will be assessed as good, moderate, or poor by pill counts (at 2nd, 3rd, and 4th visits). Participants will also be contacted daily with the methods mentioned earlier.

### Adverse events

Serious adverse events (SAE) with curcumin [19] or zinc supplementation [42] at the determined dosage have not been reported in the previous studies. However, all adverse events (AEs) including SAE and suspected unexpected serious adverse reaction (SUSAR) will be documented and reported to the Data Monitoring Committee (DMC) and the Ethics Committee of the Ahvaz Jundishapur University of Medical Sciences.

### Statistical analysis

Before statistical analysis, all data will be reviewed to check the accuracy and completeness. The primary analysis will be conducted on the intention-to-treat (ITT) population. Per-protocol (PP) and sensitivity analyses may be conducted as appropriate. The normality of data will be checked by the Kolmogorov-Smirnov test. The variables will be reported as mean ± SD or median (*Q*). The percentage changes for each variable will be separately reported and calculated by the following formula:

$$ \left[\left(E-B\right)/B\times 100\right], $$

where *E* and *B* are the end value and the baseline value of the variable, respectively.

According to the normality assumption, the significant changes between the groups will be assessed through a one-way analysis of variance (one-way ANOVA) with post hoc (LSD) analysis or Kruskal-Wallis. Changes from the baseline to post-intervention within the groups will be illustrated through a paired *t* test or Wilcoxon signed-rank test. The qualitative and quantitative variables in the two groups will be compared using the chi-square and *t* test, respectively. If necessary, the analysis of covariance (ANCOVA) will be used to control the potential confounding variables.

All analyses will be performed using a statistical software package (SPSS), version 22.0 (SPSS, Inc., Chicago, IL, USA), or NCSS2020. Statistical significance will be determined at *p* < 0.05.

### Monitoring

The present trial will be supervised and monitored by a project manager (MK) and a research advisor (HM). They will examine the trial procedures to ensure data quality and compliance with the trial protocol. The findings of the trial monitoring will be reviewed by the Data Monitoring Committee (DMC).

## Discussion

A diet rich in antioxidants [11], polyphenols [12], and phytochemicals [13] has been shown to have a beneficial role in prediabetes. According to the beneficial properties of curcumin or zinc and inadequate evidence, RCTs are needed to assess the effect of curcumin and zinc co-supplementation on glycemic measurements, lipid profiles, and inflammatory and antioxidant biomarkers in prediabetic patients. The several strengths of this trial are the evaluation of (1) the effect of zinc and curcumin co-supplementation on native prediabetic patients for the first time; (2) the adherence to co-supplementation; (3) the side effects of co-supplementation; (4) healthy lifestyle education (PA and dietary intake) weekly; (5) dietary weight-loss interventions, individually; and (6) glycemic measurements (FPG, 2hpp, HbA1c, IR, IS, BCF, and insulin), lipid profiles (TG, TC, HDL-C, LDL-C, and VLDL), inflammatory (IL-1B and hs-CRP) and antioxidant (SOD, TAC, and MDA) biomarkers, liver enzyme (ALT and AST), serum zinc, urine zinc, anthropometric measurement (weight, height, WC, HC, BMI, WHtR, WHR, FM, FFM, MM, and ABSI), dietary intake, PA, HRQOL, DBP, and SBP, simultaneously.

This study will have several limitations. First, the prediabetes diagnosis duration will not be considered. A different duration may cause more bias in the results. Short prediabetes duration at baseline was associated with a higher probability of meeting optimal care goals such as remission of prediabetes and prevents type 2 diabetes. Second, the self-reported dietary intake in this study is subject to biases. Third, self-selection bias may occur; due to the recruitment of the participants is being achieved via voluntary participation by interested subjects. These participants may be potentially cautious and more health-conscious. However, the randomization will decrease this bias. Finally, the results of this trial are not generalizable to other populations (the participants with a BMI of normal or greater than 35 kg/m^2^, children, adolescents, and the elderly), due to only recruit prediabetes adults with a 25 ≤ BMI ≤ 35 kg/m^2^.

We hope the results of the present trial, negative or positive, (1) fill this gap in the literature (the diverse recommendations are proposed to improve prediabetes such as lifestyle modification (diet plus exercise), taking herbal medicine, or taking the pharmacological drug); however, the combination therapy may be more beneficial in preventing some complications and lowering side effects, and cost-effective [57], and (2) facilitate the approach for a much larger, multi-center clinical trial.

### Trial status

The recruitment of this trial with ID number NRC-9810 on October 20, 2019, was begun in January 2020, and it seems the recruitment will be completed in December 2020 (now, it is performing).

## Supplementary Information

**Additional file 1: Table S1.** Diagnostic criteria for prediabetes. **Table S2.** The characteristics of the previous studies about the effect of curcumin and/or zinc supplement on different markers in pre-diabetes status. **Table S3.** The informed consent form.

## Data Availability

The datasets analyzed during the current study will be available from the corresponding author on reasonable request. Also, the results of this research will be published as the manuscripts in the valid databases and the Iranian Registry of Clinical Trials (IRCT) record will be updated.

## Abbreviations

ABSI: A Body Shape Index
ADA: American Diabetes Association
AEs: Adverse events
AGEs: Advanced glycation end-products
ANCOVA: Analysis of covariance
AST: Aspartate aminotransferase
ALT: Alanine aminotransferase
BCM: Beta cell function
BMC: Bisdemethoxycurcumin
BMD: Bone mineral density
BMI: Body mass index
BP: Blood pressure
CONSORT: Consolidated Standards for Reporting Trials
CUR: Curcumin
DBP: Diastolic blood pressure
DMC: Demethoxycurcumin
DLP: Dyslipidemia
FDA: Food and Drug Administration
FFM: Free fat mass
FM: Fat mass
FO: Fish oil
FPG: Fasting plasma glucose
F/M: Female/male
GRAS: Generally recognized as safe
HC: Hip circumference
HbA1c: Hemoglobin A1c
HDL-C: High-density lipoprotein cholesterol
HOMA: Homeostasis model of assessment
HRQOL: Health-related quality of life
hs-CRP: High-sensitivity C-reactive protein
IDF: International Diabetes Federation
IGT: Impaired glucose tolerance
IL-1B: Interleukin-B
IPAQ-SF: Short Form of International Physical Activity Questionnaire
IQR: Interquartile range
IRCT: Iranian Registry of Clinical Trials
IR: Insulin resistance
IS: Insulin sensitivity
LDL-C: Low-density lipoprotein cholesterol
MDA: Malondialdehyde
MET: Metabolic equivalent of task
MM: Muscle mass
MetS: Metabolic syndrome
NAFLD: Non-alcoholic fatty liver disease
One-way ANOVA: One-way analysis of variance
Pre-D: Prediabetes
pre-HTN: Prehypertension
QUICKI: Quantitative Insulin Sensitivity Check Index
RCT: Randomized clinical trial
SAE: Serious adverse events
SBP: Systolic blood pressure
SD: Standard deviation
SE: Standard error
SF-36: Short Form Health Survey
SPSS: Statistical Software Package
sUA: Serum uric acid
SUSAR: Suspected unexpected serious adverse reaction
TAC: Total blood antioxidant capacity
T/C: Treatment/control
TC: Total cholesterol
TG: Triglycerides
T2DM: Type 2 diabetes mellitus
VLDL: Very low-density lipoprotein
WC: Waist circumference
WHO: World Health Organization
WHR: Waist-hip ratio
WHtR: Waist-height ratio
2hpp: 2 h post-prandial

## References

[1] Federation I (2017). IDF diabetes atlas eighth edition.

[2] American Diabetes Association (2018). 2. Classification and diagnosis of diabetes: standards of medical care in diabetes. Diabetes Care.

[3] International Diabetes Federation (2017). Chapter 1 - What is diabetes?. IDF diabetes atlas.

[4] World Health Organization (2016). Global report on diabetes.

[5] Hostalek U (2019). Global epidemiology of prediabetes-present and future perspectives. Clin Diabetes Endocrinol.

[6] Iraj B, Salami R, Feizi A, Amini M (2015). The profile of hypertension and dyslipidemia in prediabetic subjects; results of the Isfahan Diabetes Prevention Program: a large population-based study. Adv Biomed Res.

[7] Tabák AG, Herder C, Rathmann W, Brunner EJ, Kivimäki M (2012). Prediabetes: a high-risk state for developing diabetes. Lancet.

[8] Alvarez S, Algotar AM (2017). Prediabetes.

[9] Tuso P (2014). Prediabetes and lifestyle modification: time to prevent a preventable disease. Perm J.

[10] Norris SL, Zhang X, Avenell A, Gregg E, Bowman B, Schmid CH (2005). Long-term effectiveness of weight-loss interventions in adults with pre-diabetes: a review. Am J Prev Med.

[11] van der Schaft N, Schoufour JD, Nano J, Kiefte-de Jong JC, Muka T, Sijbrands EJG (2019). Dietary antioxidant capacity and risk of type 2 diabetes mellitus, prediabetes and insulin resistance: the Rotterdam Study. Eur J Epidemiol.

[12] Guasch-Ferre M, Merino J, Sun Q, Fito M, Salas-Salvado J (2017). Dietary polyphenols, Mediterranean diet, prediabetes, and type 2 diabetes: a narrative review of the evidence. Oxidative Med Cell Longev.

[13] Abshirini M, Mahaki B, Bagheri F, Siassi F, Koohdani F, Sotoudeh G (2018). Higher intake of phytochemical-rich foods is inversely related to prediabetes: a case-control study. Int J Prev Med.

[14] Benzie IF, Wachtel-Galor S. Herbal medicine: biomolecular and clinical aspects. 2^nd^ ed. CRC Press/Taylor & Francis; 2011. 22593937

[15] Jurenka JS (2009). Anti-inflammatory properties of curcumin, a major constituent of *Curcuma longa*: a review of preclinical and clinical research. Altern Med Rev.

[16] Visioli F, De La Lastra CA, Andres-Lacueva C, Aviram M, Calhau C, Cassano A (2011). Polyphenols and human health: a prospectus. Crit Rev Food Sci Nutr.

[17] Lao CD, Ruffin MT, Normolle D, Heath DD, Murray SI, Bailey JM (2006). Dose escalation of a curcuminoid formulation. BMC Complement Altern Med.

[18] Chuengsamarn S, Rattanamongkolgul S, Phonrat B, Tungtrongchitr R, Jirawatnotai S (2014). Reduction of atherogenic risk in patients with type 2 diabetes by curcuminoid extract: a randomized controlled trial. J Nutr Biochem.

[19] Hewlings SJ, Kalman DS (2017). Curcumin: a review of its effects on human health. Foods.

[20] Azhdari M, Karandish M, Mansoori A (2019). Metabolic benefits of curcumin supplementation in patients with metabolic syndrome: a systematic review and meta-analysis of randomized controlled trials. Phytother Res.

[21] Ahangarpour A, Sayahi M, Sayahi M (2019). The antidiabetic and antioxidant properties of some phenolic phytochemicals: a review study. Diabetes Metab Syndr Clin Res Rev.

[22] Singletary K (2020). Turmeric: potential health benefits. Nutr Today.

[23] Stefanska B (2012). Curcumin ameliorates hepatic fibrosis in type 2 diabetes mellitus–insights into its mechanisms of action. Br J Pharmacol.

[24] Shehzad A, Ha T, Subhan F, Lee YS (2011). New mechanisms and the anti-inflammatory role of curcumin in obesity and obesity-related metabolic diseases. Eur J Nutr.

[25] Lee KH, Chow YL, Sharmili V, Abas F, Alitheen NB, Shaari K (2012). BDMC33, a curcumin derivative suppresses inflammatory responses in macrophage-like cellular system: role of inhibition in NF-κB and MAPK signaling pathways. Int J Mol Sci.

[26] Chuengsamarn S, Rattanamongkolgul S, Luechapudiporn R, Phisalaphong C, Jirawatnotai S (2012). Curcumin extract for prevention of type 2 diabetes. Diabetes Care.

[27] Thota RN, Acharya SH, Garg ML (2019). Curcumin and/or omega-3 polyunsaturated fatty acids supplementation reduces insulin resistance and blood lipids in individuals with high risk of type 2 diabetes: a randomised controlled trial. Lipids Health Dis.

[28] Cicero AFG, Fogacci F, Morbini M, Colletti A, Bove M, Veronesi M (2017). Nutraceutical effects on glucose and lipid metabolism in patients with impaired fasting glucose: a pilot, double-blind, placebo-controlled, randomized clinical trial on a combined product. High Blood Press Cardiovasc Prev.

[29] Yang YS, Su YF, Yang HW, Lee YH, Chou JI, Ueng KC (2014). Lipid-lowering effects of curcumin in patients with metabolic syndrome: a randomized, double-blind, placebo-controlled trial. Phytother Res.

[30] Amin F, Islam N, Anila N, Gilani A (2015). Clinical efficacy of the co-administration of turmeric and black seeds (Kalongi) in metabolic syndrome–a double blind randomized controlled trial–TAK-MetS trial. Complement Ther Med.

[31] Rahmani S, Asgary S, Askari G, Keshvari M, Hatamipour M, Feizi A (2016). Treatment of non-alcoholic fatty liver disease with curcumin: a randomized placebo-controlled trial. Phytother Res.

[32] Grant SJ, Chang DH-T, Liu J, Wong V, Kiat H, Bensoussan A (2013). Chinese herbal medicine for impaired glucose tolerance: a randomized placebo controlled trial. BMC Complement Altern Med.

[33] Chasapis CT, Loutsidou AC, Spiliopoulou CA, Stefanidou ME (2012). Zinc and human health: an update. Arch Toxicol.

[34] Chausmer AB (1998). Zinc, insulin and diabetes. J Am Coll Nutr.

[35] Jarosz M, Olbert M, Wyszogrodzka G, Mlyniec K, Librowski T (2017). Antioxidant and anti-inflammatory effects of zinc. Zinc-dependent NF-κB signaling. Inflammopharmacology.

[36] Jayawardena R, Ranasinghe P, Galappatthy P, Malkanthi R, Constantine G, Katulanda P (2012). Effects of zinc supplementation on diabetes mellitus: a systematic review and meta-analysis. Diabetol Metab Syndr.

[37] Ranasinghe P, Wathurapatha W, Ishara M, Jayawardana R, Galappatthy P, Katulanda P (2015). Effects of zinc supplementation on serum lipids: a systematic review and meta-analysis. Nutr Metab.

[38] Lukowiak B, Vandewalle B, Riachy R, Kerr-Conte J, Gmyr V, Belaich S (2001). Identification and purification of functional human β-cells by a new specific zinc-fluorescent probe. J Histochem Cytochem.

[39] Islam MR, Attia J, Ali L, McEvoy M, Selim S, Sibbritt D (2016). Zinc supplementation for improving glucose handling in pre-diabetes: a double blind randomized placebo controlled pilot study. Diabetes Res Clin Pract.

[40] Ranasinghe P, Wathurapatha WS, Galappatthy P, Katulanda P, Jayawardena R, Constantine GR (2018). Zinc supplementation in prediabetes: a randomized double-blind placebo-controlled clinical trial. J Diabetes.

[41] Kim H-N, Kim S-H, Eun Y-M, Song S-W (2018). Effects of zinc, magnesium, and chromium supplementation on cardiometabolic risk in adults with metabolic syndrome: a double-blind, placebo-controlled randomised trial. J Trace Elem Med Biol.

[42] Bogale A, Clarke SL, Fiddler J, Hambidge KM, Stoecker BJ (2015). Zinc supplementation in a randomized controlled trial decreased ZIP4 and ZIP8 mRNA abundance in peripheral blood mononuclear cells of adult women. Nutr Metab Insights.

[43] Lotfi MH, Saadati H, Afzali M (2013). Prevalence of diabetes in people aged ≥30 years: the results of screening program of Yazd Province, Iran, in 2012. J Res Health Sci.

[44] Pavlica S, Gaunitz F, Gebhardt R (2009). Comparative in vitro toxicity of seven zinc-salts towards neuronal PC12 cells. Toxicol in Vitro.

[45] Energy requirements of adults. In: FAO food and nutrition technical report series. Human energy requirements: report of a joint FAO/WHO/UNU expert consultation. Food Nutr Bull. 2005; 26(1):35–50. 15810802

[46] Knowler WC, Barrett-Connor E, Fowler SE, Hamman RF, Lachin JM, Walker EA, Diabetes Prevention Program Research Group (2002). Reduction in the incidence of type 2 diabetes with lifestyle intervention or metformin. N Engl J Med.

[47] Acceptable macronutrient distribution ranges. In: Otten J. Hellwig J. Meyer L. Dietary Reference Intakes. Washington, DC: National Academies Press; 2006. p. 70.

[48] Krakauer NY, Krakauer JC (2012). A new body shape index predicts mortality hazard independently of body mass index. PLoS One.

[49] Pickering T, Hall J, Appel L, Falkner B, Graves J, Hill M (2005). Recommendations for blood pressure measurement in humans. A statement for professionals from the subcommittee of Professional and Public Education of the American Heart Association Council on High Blood Pressure Research. Hypertension.

[50] Rifai N, Bachorik PS, Albers JJ (1999). Lipids, lipoproteins and apolipoproteins. Tietz Textbook Clin Chem.

[51] Yang YJ, Kim MK, Hwang SH, Ahn Y, Shim JE, Kim DH (2010). Relative validities of 3-day food records and the food frequency questionnaire. Nutr Res Pract.

[52] Vasheghani-Farahani A, Tahmasbi M, Asheri H, Ashraf H, Nedjat S, Kordi R (2011). The Persian, last 7-day, long form of the International Physical Activity Questionnaire: translation and validation study. Asian J Sports Med.

[53] Craig CL, Marshall AL, Sjöström M, Bauman AE, Booth ML, Ainsworth BE (2003). International Physical Activity Questionnaire: 12-country reliability and validity. Med Sci Sports Exerc.

[54] Montazeri A, Goshtasebi A, Vahdaninia M, Gandek B (2005). The Short Form Health Survey (SF-36): translation and validation study of the Iranian version. Qual Life Res.

[55] Osoba D (1999). What has been learned from measuring health-related quality of life in clinical oncology. Eur J Cancer.

[56] Framework IC (1992). The MOS 36-item short-form health survey (SF-36). Med Care.

[57] Kanat M, DeFronzo RA, Abdul-Ghani MA (2015). Treatment of prediabetes. World J Diabetes.

