# Supplementary material for: Evaluation of the effect of curcumin and zinc co-supplementation on glycemic measurements, lipid profiles, and inflammatory and antioxidant biomarkers in overweight or obese prediabetic patients: a study protocol for a randomized double-blind placebo-controlled phase 2 clinical trial
Source: Trials. 2020 Nov 30;21:991. doi: 10.1186/s13063-020-04923-w (PMC7708225; doi:10.1186/s13063-020-04923-w)
Supplement: Supplementary file 1 — Additional file 1: Table S1. Diagnostic criteria for prediabetes. Table S2. The characteristics of the previous studies about the effect of curcumin and/or zinc supplement on different markers in pre-diabetes status. Table S3. The informed consent form. [file 13063_2020_4923_MOESM1_ESM.doc]

**Evaluation of the effect of curcumin and zinc co-supplementation on glycemic measurements, lipid profiles, inflammatory and antioxidant biomarkers in overweight or obese pre-diabetic patients: a study protocol for a randomized double-blind placebo-controlled phase 2 clinical trial**

**Majid Karandish^1^, Hassan Mozaffari-khosravi^2^, Seyed Mohammad Mohammadi^3^, Bahman Cheraghian^4^, Maryam Azhdari ^5, 6*^**

^1^Nutrition and metabolic Diseases Research Center, Ahvaz Jundishapur University of Medical Sciences, Ahvaz, Iran.

^2^ Department of Nutrition, School of Public Health, Shahid Sadoughi University of Medical Sciences, Yazd, Iran.

^3^ Associate Professor of Endocrinology & Metabolism, School of Medicine, Shahid Sadoughi University of Medical Sciences, Yazd, Iran.

^4^ Department of Biostatistics and Epidemiology, School of Health Sciences, Ahvaz Jundishapur University of Medical Sciences, Ahvaz, Iran.

^5^ Department of nutrition, School of Allied Medical Sciences, Ahvaz Jundishapur University of Medical Sciences, Ahvaz, Iran.

^6^ Student Research Committee, Ahvaz Jundishapur University of Medical Sciences, Ahvaz, Iran.

***Corresponding Author:**

Maryam Azhdari

Department of nutrition, School of Allied Medical Sciences, Ahvaz Jundishapur University of Medical Sciences, Ahvaz, Iran.

Student Research Committee, Ahvaz Jundishapur University of Medical Sciences, Ahvaz, Iran.

Tel: +989131571909 Tel: +986133738383

Email: [azhdari_mar@yahoo.com](mailto:azhdari_mar@yahoo.com)

Orcid ID: 0000-0003-2110-9817

**Supplementary material**

**Table S.1:** Diagnostic criteria for prediabetes.

|  | **IFG** | **IGT** | **HbA1C** |
| --- | --- | --- | --- |
| **ADA** | FPG ≥ 5.6 and < 6.9 mmol/L (≥100 and <126 mg/dl) | 2-h PG* ≥7.8 and < 11.0 mmol/L (≥140mg/dl and < 200mg/dl) | 5.7–6.4%  (39-47 mmol/mol) |
| **IDF** | FPG ≥ 6.1 and < 6.9 mmol/L (≥110 and <126 mg/dl)  **or**  2-h plasma glucose < 7.8mmol/L (140mg/dL) | FPG <7.0 mmol/L (126mg/dl)  **and**  2-h PG ≥7.8 and < 11.0 mmol/L (≥140mg/dl and < 200mg/dl) | - |
| **WHO** | FPG ≥ 6.1 and < 6.9 mmol/L (≥110 and <126 mg/dl)  **and (if measured)**  2–h plasma glucose < 7.8 mmol/ L (<140mg/dL) | FPG <7.0 mmol/L (126mg/dl)  **and**  2-h PG ≥7.8 and < 11.0 mmol/L (≥140mg/dl and < 200mg/dl) | - |

IFG: Impaired Fasting Glucose; IGT: Impaired Glucose Tolerance; HbA1C: glycated hemoglobin A1C;

ADA: American Diabetes Association; IDF: International Diabetes Federation; WHO: World Health Organization;

*following a 75g oral glucose load

**Table S.2: The characteristics of the previous studies about the effect of curcumin and/or zinc supplement on different markers in pre-diabetes status.**

| **Reference** | **Design (location, duration) & participants** | | **Study arms** | **Co-treatment** | **Assessed Outcomes** |
| --- | --- | --- | --- | --- | --- |
| **Curcumin** | | | | | |
| Chuengsamarn et al. (2012) | RP, DB (China, 9 months)  pre-D (ADA criteria)  F/M  N=237, T/C: (120/117) | | T: CC capsule (curcuminoids extract 1.5 g/day)  C: Placebo | Healthy lifestyle education, no medication | HbA1c, FPG, 2HPP, BCF, IR, BMD, liver enzymes, creatinine, adiponectin, and AEs |
| Grant SJ et al. (2013) | RP, DB (Australia, 16 weeks)  Pre-D or controlled T2DM  F/M  N= 71: T/C (39/32) | | T: herbal Combination (Curcuma longa=1.5g/day)  C: Placebo | Routine lifestyle | FBG, 2 hr OGTT and HbA1c, insulin, IR, IS, TC, TG, HDL-C, CRP, BMI, waist girth, BP, HRQoL, and AEs. |
| Yang et al. (2014) | RP, DB (Thailand, 6 months)  MetS (Pre-D, pre-HTN, DLP)  F/M  N=65 T/C: (33/32) | | T: CC capsule (curcuminoids extract 95% 1.9 g/day = 1.8 g/day)  C: Placebo | Routine lifestyle | HbA1c, FPG, TG, TC, LDL, HDL, VLDL, non-HDL, TC/ HDL ratio, weight, BMI, and |
| Amin et al. (2015) | RP, DB (Pakistan, 8 weeks)  MetS (Pre-D, pre-HTN, DLP)  M  N= 250 T/C:(turmeric:63,turmeric-black seeds 62,black seeds 62/63) | | T: Turmeric powder in capsule (2.4 g/day)  C: Placebo  Other groups: Black seeds and combination | Healthy lifestyle education, no medication | FPG, TG, TC, LDL, HDL, WC, HC, BMI, BP, CRP, AEs |
| Rahmani et al. (2016)  ( | RP, DB (Iran, 8 weeks)  MetS (Pre-D, pre-HTN, DLP), NAFLD  F/M  N=80 T/C:(40/40) | | T: CC capsule (curcuminoids 70 mg/day)    C: Placebo | No medication | HbA1c, FPG, TG, TC, LDL, HDL, weight, BMI, liver enzymes, NAFLD severity, and AEs . |
| Cicero AFG et al. (2017) | RP, DB (Italy, 8weeks)  IFG  F/M  N= 40: T/C (20/20) | | T: nutraceutical Combination (Curcuma extract 125 mg)  C: Placebo | Healthy lifestyle education, | Weight, WC and BMI, BP, cardiac frequency, TC, HDL-C, TG, LDL-C, non HDL-C, FPG, fasting insulin, sUA, hsCRP, and AEs. |
| Thota RN et al.  (2019) | RP, DB (Australia, 12weeks)  IFG, IGT  F/M  N= 64: T/C (CC:15, CC-FO: 16, FO:17/16) | | T: CC ( 180 mg/day)  Other groups: FO and combination  C: Placebo | Routine lifestyle | Weight, muscle mass, BMI, body fat%, WC, HbA1c, FBG, serum insulin, IR, TC, TG, HDL-C, LDL-C, TC: HDL-C, CRP, whole blood cell count, Dietary intake, PA, AEs. |
| **ZINC** | | | | | |
| Islam MR et al.  (2016) | RP, DB (Bangladesh, 6months)  Pre-D  F/M  N= 55: T/C (28/27) | T: zinc sulphate (zinc:30 mg)  C: Placebo | | Healthy lifestyle education, | FPG, IR, IS, BCM, HbA1c, serum insulin, Serum zinc, TG, HDL, LDL, CRP, and AEs. |
| Ranasinghe P et al.  (2018) | RP, DB (South Korea, 12months)  Pre-D  F/M  N= 200: T/C (100/100) | T: zinc capsule (elemental zinc: 20 mg/day )  C: Placebo | | - | Height, weight, BMI, WC, HC, WHR, FPG, 2h OGTT, SBP, DBP, TC, LDL-C, HDL-C,TG, AST, ALT, Serum bilirubin, Serum creatinine, serum zinc, PA and AEs. |
| Kim HN et al.  (2018) | RP, DB (South Korea, 24 weeks)  MetS  F/M  N= 32: T/C (16/16) | T: zinc oxide (elemental zinc 36 mg/day)  Other combination: Mn, Cr  C: Placebo | | Routine lifestyle | HDL-c, TG, FBG, BP, WC, IR, CRP. |

ADA, American Diabetes Association; AEs, adverse events; AST, Aspartate aminotransferase; ALT, Alanine aminotransferase; BCM, beta cell function; BMD, bone mineral density; BMI, body mass index; BP, blood pressure; CC: Curcumin; DB, double-blinding; DLP, dyslipidemia; FO, fish oil; FPG, fasting plasma glucose; F/M, female/male; HC, hip circumference; HbA1c, hemoglobin A1c; HDL-C, high-density lipoprotein cholesterol; IR, insulin resistance; IS, LDL-C, low-density lipoprotein cholesterol; Mn, magnesium, Cr, chromium; MetS, metabolic syndrome; NAFLD, non-alcoholic fatty liver disease; Pre-D, prediabetes; pre-HTN, prehypertension; RP, randomized parallel design; sUA: Serum Uric Acid; T/C, treatment/control; T-Chol, total cholesterol; TG, triglycerides;T2DM, type 2 diabetes mellitus; VLDL, very low-density lipoprotein cholesterol; WC, waist circumference; WHR: waist: hip ratio; 2-hours postprandial glucose

**Table S.3.** The informed consent form.

| \| 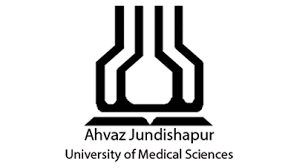 *In the name of God* \| 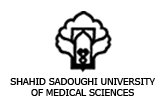 \| \| --- \| --- \|   ***Informed Parental Consent for Research Involving Children/ informed consent for the students* *entitled*** *“Evaluation of the effect of curcumin and zinc co-supplementation on glycemic measurements, lipid profiles, inflammatory and antioxidant biomarkers in overweight or obese pre-diabetic patients: a study protocol for a randomized double-blind placebo-controlled phase 2 clinical trial”.*  I have read the foregoing information, or it has been read to me. I have had the opportunity to ask questions about it and any questions I have been asked have been answered to my satisfaction. I consent voluntarily to be a participant or allow my children to be a participant in this study.    **Print Name of Participant __________________**  **Signature of Participant ___________________**  **Date ___________________________ Day/month/year**  ***If illiterate ^[[1]](#footnote-1)^***  I have witnessed the accurate reading of the consent form to the potential participant, and the individual has had the opportunity to ask questions. I confirm that the individual has given consent freely.  **Print name of witness____________ Thumbprint of participant**  **Signature of witness _____________**  **Date ________________________ Day/month/year**  **Statement by Maryam Azhdari**  I have accurately read out the information sheet to the potential participant, and to the best of my ability made sure that the participant understands that the following will be done:   1. Recommendation to take two supplements (a tablet as zinc supplement and a capsule az curcumin supplement) 2. Collecting demographic and medical information (gender, age, full address, postal code, marital status, income, occupation, ethnicity, educational background, smoking history, alcohol consumption, family history of diabetes, family history of hypertension, medication history (dosage and type of drugs), and the diagnosis time of pre-diabetes) and (only at baseline of the study) 3. Recording Adverse events and Participant adherence to the intake of the supplements 4. Estimating health-related quality of life (HRQOL) by Short Form Health Survey (SF-36) questionnaire (at the baseline and the end of the study (day 90 after intervention)). 5. Estimating physical activity by Short form of International Physical Activity Questionnaire (IPAQ-SF) (at the baseline and the end of the study (day 90 after intervention)). 6. Estimating daily food intake by a 3-day food intake records (2 weekdays, 1 weekend day) (at the baseline and the end of the study (day 90 after intervention)). 7. Measuring Anthropometric data (weight, height, waist circumstance (WC), hip circumstance (HC), body mass index (BMI), waist-height ratio (WHtR), waist-hip ratio (WHR), fat mass (FM), free fat mass (FFM), muscle mass (MM), and A Body Shape Index (ABSI)) and Blood Pressure (at the baseline and the end of the study (day 90 after intervention)). 8. Colleting 12 ml-blood sample for measuring FBG, 2h-OGTT, HbA1c, serum insulin, IR, IS, BCF, TG, TC, LDL-C, HDL-C, TAC, MDA, serum zinc, urine zinc, IL-1B, hs-CRP,ALT, AST (at the baseline and the end of the study (day 90 after intervention)). 9. Collecting 1 ml-urine specimen for measuring urine zinc (at the baseline and the end of the study (day 90 after intervention)).   I confirm that the participant was given an opportunity to ask questions about the study, and all the questions asked by the participant have been answered correctly and to the best of my ability. Also, I ensure that participants receive good clinical care and that safety concerns are identified quickly and addressed appropriately. The executors of the present project review each AE/SAE and consider whether it may be related to study participation. All costs related to unusual/ unnecessary diagnostic or therapeutic methods (predicted or unpredictable) during this project will be borne by the research team and the patient will not pay a fee.  I confirm that the individual has not been coerced into giving consent, and the consent has been given freely and voluntarily.     A copy of this ICF has been provided to the participant.    **Maryam Azhdari**  **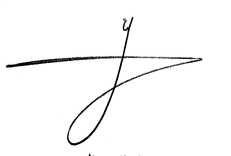Date ___________________________ Day/month/year** |
| --- | --- | --- |

1. A literate witness must sign (if possible, this person should be selected by the participant and should have no connection to the research team). Participants who are illiterate should include their thumb print as well. [↑](#footnote-ref-1)
